# Characterization of Composite Edible Films Based on Pectin/Alginate/Whey Protein Concentrate

**DOI:** 10.3390/ma12152454

**Published:** 2019-08-01

**Authors:** Swathi Sirisha Nallan Chakravartula, Michela Soccio, Nadia Lotti, Federica Balestra, Marco Dalla Rosa, Valentina Siracusa

**Affiliations:** 1 Department of Agricultural and Food Sciences- DISTAL, University of Bologna, Campus of Food Science, P.zza Goidanich 60, 47521 Cesena, Italy; 2 Department of Civil, Chemical, Environmental and Materials Engineering, University of Bologna, Via Terracini 28, 40131 Bologna, Italy; 3 Department of Chemical Science, University of Catania, Viale A. Doria 6, 95125 Catania (CT), Italy

**Keywords:** biopolymers, composite film, physico-chemical properties, thermal characteristics, barrier properties, response surface

## Abstract

Edible films and coatings gained renewed interest in the food packaging sector with polysaccharide and protein blending being explored as a promising strategy to improve properties of edible films. The present work studies composite edible films in different proportions of pectin (P), alginate (A) and whey Protein concentrate (WP) formulated with a simplex centroid mixture design and evaluated for physico-chemical characteristics to understand the effects of individual components on the final film performance. The studied matrices exhibited good film forming capacity, except for whey protein at a certain concentration, with thickness, elastic and optical properties correlated to the initial solution viscosity. A whey protein component in general lowered the viscosity of the initial solutions compared to that of alginate or pectin solutions. Subsequently, a whey protein component lowered the mechanical strength, as well as the affinity for water, as evidenced from an increasing contact angle. The effect of pectin was reflected in the yellowness index, whereas alginate and whey protein affected the opacity of film. Whey protein favored higher opacity, lower gas barrier values and dense structures, resulting from the polysaccharide-protein aggregates. All films displayed however good thermal stability, with degradation onset temperatures higher than 170 °C.

## 1. Introduction

Environmentally friendly technology aimed at food safety, quality and easy handling properties have promoted a huge interest for the research in edible films and coatings for applications in food and food packaging, due to their bio-degradability, natural protection from the external environment and potential use as carrier systems for active substances, such as antioxidants, antimicrobials, flavoring, and/or coloring agents [1,2,3]. The terms edible films and coatings are defined by their method of application: Films are applied after being formed and coatings are formed directly onto the product surface [2]. Edible film, in particular, is defined as a thin layer(s) of edible polymers formed on food surfaces or wrapped around food products as primary packaging, and generally with supplementary benefits. As extensively reviewed, such systems can could be used to limit moisture migration, to reduce lipid migration, and they have found various applications in the conservation of highly perishable products (especially fresh and minimally processed fruits and vegetables), for semi-perishable products like processed meats (sausages) and cheese and, also, as barrier materials to reduce oil absorption in fried foods [4,5,6,7]. These systems could be used to reduce the food senescence processes, avoiding the worsening of the visual appearance of coated commodities. Mechanical (strength and flexibility), thermal, optical (brightness and opacity), wettability and morphological properties are strongly influenced from coating and film composition. Therefore, preparation conditions, such as solvent, pH, component concentration and temperature, and types of additives, such as cross-linking agents, antimicrobials, plasticizers and emulsifiers, must be carefully controlled.

Edible coatings and films require several components to be formulated with desired structural properties. Polysaccharides, protein and hydrocolloid compounds, derived from food extraction and plant tissue, are among them. Polysaccharides are characterized by poor gas and water barrier properties, usually acting as sacrificing agents for moisture loss [8]. Proteins are known for their ability to form film, similar to that of polysaccharides with excellent mechanical and barrier properties, although they make poor water vapor barriers. Literature suggests several methods to improve the properties of edible films. One approach being blending polysaccharides and proteins. This is an area of great interest in the field of material science, to develop new functionalities using well characterized components or totally new components [9,10]. As an example, water barrier efficiency is important to decrease dehydration in fresh food, as well as to prevent loss of crispiness and moisture migration in dry food [7].

Pectin is an anionic polysaccharide present in most plant tissues and it is made up of 1,4-D-galacturonic acids with fully or partially methyl esterified carboxyl groups [11]. It is the major by-product of the fruit and vegetable industry and it is widely studied for edible film formulation [12,13,14]. However, Pectin film is characterized by high water vapor permeability, acting as a sacrificial agent to prevent dehydration.

Alginates are structural polysaccharides made up of alternating blocks of mannuronic acid (M) and glucuronic acid (G) residues, extracted from brown algae [15]. These ionic linear co-polymers are known as polyuronates and, under suitable pH conditions, soluble solids form synergistic gels with pectin. Consequently, they are also studied in the preparation of edible film [12].

Whey protein is a by-product of dairy industry, which has received great attention from the nutrition and polymer industries, in form of whey protein concentrates and whey protein isolates containing from 20% to more than 90% of protein, respectively. They have been observed to possess excellent mechanical and barrier properties, as reviewed by various researchers [3,16,17,18]. Many studies investigated the potential use of whey protein isolates and concentrates to form edible films and coatings [19]. Whey protein films are transparent, flexible and have good water, gas, aroma and oil barrier characteristics. Thermal degradation denatures whey protein, resulting in a more cohesive and stronger film than native proteins [20].

In order to design new materials with improved and selective properties, it is important to study and understand their compatibility. Consequently, several composite films can be designed to achieve synergistic effects of the pure components, once the characteristics and compatibility of the individual components have been determined. As reported in literature, blends of two or more hydrocolloids can result in improved film properties, reflecting or not the properties of individual components [12,21,22]. The optimization of composition is one of the most important steps to be considered in the edible film formulation, to maintain or improve the quality of food packed. Pectin, alginates and whey proteins are some of the widely investigated components for film development singularly, as well as in two-way interactions.

Considering the synergistic effects of pectin–alginate or alginate–whey protein interactions, the objective of this study was to investigate the final properties of edible films based on polysaccharides and polysaccharides-proteins, in particular to understand the interaction of pectin-alginate-whey protein concentrate to form edible films. For this, different pectin-alginate-whey protein concentrate blend solutions were conceived by use of simplex centroid design and further investigated. Chemical, physical and structural analyses have been performed on the prepared solutions in order to evaluate the corresponding properties of interest for the in-use applications, such as coating, as well as for edible film. These compositions were investigated for potential application in bakery products, like mini-burger buns [23], bread and biscuits, in order to extend the shelf life, and also as potential matrices to incorporate functional characteristics like probiotics, antimicrobials or antioxidant compounds [24,25]. In particular, the drying process of those materials was studied by near infrared spectroscopy (NIR) and by a heat and mass transfer numerical model, on mini-burger buns [23,26].

## 2. Materials and Methods

### 2.1. Materials

The raw materials used for the preparation of films were pectin (from citrus peel, Galacturonic acid ≥74%, Sigma, Denmark), Sodium alginate (Sigma Aldrich, China) and whey protein concentrate (protein ≅ 80%, Prodotti-Gianni, Italy). Glycerol (≥99.5% purity, Sigma Aldrich, Germany) and Tween^®^20 (Sigma Aldrich, Germany) were used as plasticizer and emulsifier, respectively. All materials were used as received, without any further purification.

### 2.2. Experimental Design and Analysis

An augmented simplex centroid mixture design was used to find the optimum combination of constituents, using Statistica (Version 8.0). The 10 runs obtained, as reported in Table 1, were coded as S_1–10_ (P:A:WP), where ‘S’ represents sample, the numbers ‘1–10’ are self-evident, and the different formulations and P:A:WP represent the different combinations of pectin (P), alginate (A), and whey protein concentrate (WP). The proportions of components are dependent on each other, and hence the selection of design points is based on the basic constraint for the mixture design as in Equation (1):

(1) $$\overset{n}{\underset{i=1}{\sum }}x_{i\;}=x_{1}+x_{2}\;+\;\ldots \;+\;x_{n}=1$$

where *x_i_* is the proportion of ‘*i^th^* ’component in the mixture and ‘*n*’ is the number of components in the mixture. From the preliminary experiments, pectin, alginate and whey protein concentrate were chosen as input factors, with their proportions restricted to a total sum of ‘3’ for actual values by weight; i.e.,:

- 0 ≤ P (Pectin) ≤ 3
- 0 ≤ A (Alginate) ≤ 3
- 0 ≤ WP (Whey Protein Concentrate) ≤ 3

The concentrations of glycerol and Tween^®^20 were determined from preliminary experiments to a constant of 1% w/w and 0.1% w/w concentrations, respectively. Water was considered as a bulk agent added at 95.9 g to make up 100 g of total weight of the solution and was not included in the design.

The regression coefficients (β’s), for each response (Y’s), were estimated by polynomial models to screen the selected responses, to understand component interactions. The models were subjected to analysis of variance (ANOVA) to assess the level of significance, the coefficient of determination (R^2^) and the lack of fit of the model. The program Statistica 7.0 (StatSoft, 2004) was used to generate the experimental design and regression coefficients, as well as for construction of the graphs**.** The analysis of variance (ANOVA) with Tukey HSD (honestly significant difference) for homogenous variances and Kruskall-Wallis tests for non-homogenous variances were performed to detect significant differences in properties of films at a significance level of 0.05.

### 2.3. Film Formation Solutions (FFS) and Edible Film Preparation

The components used for each composition, as reported in Table 1, were weighed, dispersed in water (95.9 g to 3 g of biopolymer) and heated on a heating plate with magnetic stirrer (ARE Heating Magnetic stirrer, Velp Scientific) at 75 °C ( ± 0.1 °C) for 45 min, until complete dissolution. The obtained solutions were cooled to room temperature, homogenized at 6000 rpm for one minute (IKA Ultra Turrax^®^ T 25 Digital, Germany) and degassed under vacuum (100 mb) with a pump (SC 920, KNF ITALIA, Milano) for ten minutes [27]. Subsequently 10 ± 1 g of solutions were plated in petri-dishes (90 mm diameter) and dried for 18–24 h at 25 ± 1 °C and 50 ± 2% of relative humidity, in a controlled atmospheric chamber (Constant Climate Chamber with Peltier technology, model HPP 108/749—Memmert, Schwabach, Germany), to ensure a complete solvent removal. The dried films were detached from the petri-plates carefully by peeling and conditioned in controlled atmospheric chamber (Constant Climate Chamber with Peltier technology, model HPP 108/749- Memmert, Germany) at 25 ± 1 °C and 50 ( ± 2)% RH (relative humidity) for 48 h prior to testing moisture and water vapor permeability. Subsequently, the remaining dried films were stored in a desiccator (with silica gel) prior to testing mechanical, thermal and other barrier parameters.

### 2.4. Film Forming Solutions Characterization

The pH of the film-forming solutions (FFS) were measured at room temperature by digital pH meter (EUTECH Instruments, Cyberscan mod.510, D, Italy), while the Total Soluble Solid (T.S.S, Brix) was determined by digital refractometer (Atago Co. Ltd., mod. PR-1, Tokyo, Japan). Density was calculated as ratio of weight to volume (g/mL) with an analytical weighing balance (Kern & Sohn, model: ABJ 220-4M, Germany) to the nearest 0.0001 g, according to Zhong et al. [28].

Rheological measurements were performed at 25 °C, with a stress-strain rheometer (model: MCR 102, Anton Paar GmbH, Inc., Graz, Austria), using a coaxial cylinder-CC27 apparatus. Samples of 20 mL in duplicates were deposited carefully into the sample holder and pre-sheared at 10 s^−1^ and rested for 60 s for equilibration. Steady-state flow measurements were carried out in the range of 3 s^−1^ to 500 s^−1^ with a rate of 10 s^−1^/min in 30 min. The resulting flow curves (viscosity-shear rate) were fitted using Carreau model [29] in order to obtain parameters ‘a’ (time constant, dimensionless) and ‘p’ (dimensionless exponent) and by using the power-law model [11,30] to obtain parameters ‘k’ (consistency index, Pa.s^−n^) and ‘n’ (flow behavior index, dimensionless), respectively.

### 2.5. Edible Film Characterization

#### 2.5.1. Optical Parameters

The color of the film samples was measured using a HunterLab ColorFlex EZ 45/0° TM (mod. A60-1010-615) color spectrophotometer (Hunterlab, Reston, VA, USA), with the D65 illuminant, 10° observer. The measurements were made using the CIE Lab scale, according to ASTM E308 (*Practice For Computing The Colors Of Objects By Using The CIE System*). The results are expressed as L* (luminosity), a* (red/green) and b* (yellow/blue) parameters. The total color difference (∆E), whiteness index (WI) and yellowness index (YI) were calculated as described in the literature [31,32]. The instrument was calibrated with black and white standard tiles. The white standard tile, with L* = 93.47, a* = −0.83 and b* = 1.33, was used as background for color measurements. The total color difference (∆E) was calculated according to the following Equation (2):

∆E = [(L* − L’)^2^ + (a* − a’)^2^ + (b* − b’)^2^]^1/2^ (2)

where L*, a* and b* are the color parameter values of the sample, and L’, a’ and b’ are the color parameter values of the standard white plate used as the film background (L’ = 66.39, a’ = −0.74, b’ = 1.25). A mean value recorded for the top side and bottom side is reported. The analyses were conducted in five repetitions.

The opacity of the films was calculated as ratio of absorbance (A) (in nm) to the film thickness (in μm) using an UV-VIS spectrophotometer (Shimadzu Italia srl, mod. UV-1601, Milano, Italy) according to Galus and Kadzinska experiments [17]. Rectangular film strips (1 cm × 4 cm) were placed directly in glass cuvettes (UV/Vis Helios Gamma spectrophotometer test cell, Thermo Fisher Scientific, Waltham, MA, USA), using an empty glass cuvette as reference. The absorbance was measured at 600 nm and the opacity of the film was calculated by the following, Equation (3):

O= A_600nm_/δ (3)

where O = opacity; A_600nm_ = absorbance of the sample at 600nm; δ = Thickness of the film in mm

The analyses were conducted in five repetitions and a mean value was reported.

#### 2.5.2. Microstructure

The film structural morphology was determined by using a Nikon upright microscope (Eclipse Ti-U, Nikon Co., Shanghai, Japan) with a standard light. Samples were observed in black and white and the images were recorded at ×20 magnification. The analyses were conducted for five repetitions, at room temperature, and a mean value was reported.

#### 2.5.3. Thickness and Mechanical Properties

Film thickness (δ) was measured using a Sample Thickness Tester DM-G with a digital dial indicator (model MarCartor 1086 type, Mahr GmbH, Esslingen, Germany) with associated DM-G software. The reading was made twice per second, with a resolution of 0.001 mm. The minimum, maximum and average of each reading was recorded in triplicates, in 10 different positions of each film, at room temperature and reported as mean thickness value, expressed in microns.

Mechanical properties were evaluated by using a Texture Analyser (ModZ2.5, Zwick Roell, Ulm, Germany) equipped with a rubber grip and a 500 N load cell, in accordance with ASTM D882-09 (*Standard test method for Tensile properties of Thin plastic sheeting, 2009*). Rectangular strips (5 mm width × 50 mm length) were loaded with an initial grip separation of 20 mm and crosshead speed of 5 mm/min. A pre-load of 1 MPa with a pre-load speed of 5 mm/min was applied. Tensile strength (σ^B^, MPa), Elongation at break (ε^B^, %) and Elastic modulus (E, MPa/%) was evaluated on six different samples and the results were provided as the average value ± standard deviation.

#### 2.5.4. Moisture

The moisture content was measured according to Galus and Lenart [12], with 1.5 × 1.5 cm films weighed to the nearest 0.0001 g and dried in hot air oven (UF110, Memmert, Schwabach, Germany), at 105 °C, for 4 h in triplicates, and calculated as percent loss in the weight compared to that of initial sample weight.

#### 2.5.5. Water Contact Angle

Static water contact angle (θ) of the films was measured by sessile drop method using a KSV CAM101 (KSV Instrument, Elsinki, Finland) instrument, at room temperature [32]. The side profile of deionized water drops (4 μL) deposited on the film surface was measured. The measurement was taken 2 s after the drop deposition to ensure its stabilization and yet to minimize the water absorption and evaporation. The measurements were carried out on both sides of the films and reported as the average of at least 5 individual determinations on each side. Contact angle data, expressed in °, were reported as the average value ± standard deviation.

#### 2.5.6. Water Vapor Barrier Properties

The water vapor transmission rate (WVTR) of films was measured in accordance with ASTM E-96-95 (*Standard Test Method for Water Vapor Transmission of Materials, 1995*), following the methodology described by Cecchini et al. [33], with some modifications. In brief, glass cups with a cylindrical base were deposited with 4 g of CaCl_2_. The support side of the film was fixed onto the opening side so as to face the low RH environment, fixed with paraffin and placed in a pre-equilibrated atmospheric chamber (Constant Climate Chamber with Peltier technology, model HPP 108/749- Memmert, Schwabach, Germany) at 25 ± 1 °C and 50 ± 2 % RH The weight of the assembled cups was periodically recorded for 72 h until CaCl_2_ was visibly wet. The water vapor transfer rates were determined from slope of weight gain versus time plots for each sample, according to Equation (4):

(4) $$WVTR\;\left(g\;cm^{-2}h^{-1}\right)=\frac{\Delta m}{\Delta t*A}$$

where, Δm is weight gain of the test setup (g), Δt is time (h) and A is the area of film exposed which was of 23.76 cm^2^. Water vapor permeability (WVP) was calculated by the following Equation (5):

(5) $$WVP\;\left(g\;mm\;cm^{-2}Pa^{-1}h^{-1}\right)=\;WVTR*\;\frac{x}{\Delta P}$$

where, *x* is the average thickness of the films in mm and ΔP is partial difference of water vapor between the 50% RH of the chamber and 0% RH of CaCl_2_, which was 1579.09 Pa. All experiments were done in triplicate and mean value was reported.

#### 2.5.7. Gas Barrier Properties

Oxygen and carbon dioxide gas barrier properties were measured using a manometric method on a permeance testing device, type GDP-C (Brugger Feinmechanik GmbH, Munchen, Germany), in accordance with the ASTM 1434 (*standard test method for determining gas permeability characteristics of plastic film and sheeting, 2009*), DIN 53536 (*gas permeability determination*), ISO 15105-1(*Plastics film and sheeting determination of gas transmission rate—Part I: Differential pressure methods, 2007*) and with the Gas Permeability Testing Manual, 2008 (Registergericht München HRB 77020, Brugger Feinmechanik GmbH) [32]. The film sample of approximately 3 cm × 3 cm in size was mounted between the two chambers of the equipment. A film mask was used to cover the remaining section of the permeation chamber [32]. The top chamber was filled with the dry test gases at ambient pressure and subsequent gas permeation was determined by evaluating the pressure increase in the evacuated bottom chamber. Test conditions used were: 23 °C, gas stream of 100 cm^3^/ min with 0% of Gas RH; sample area of 0.785 cm^2^. The temperature was regulated by an external thermostat (HAAKE-circulator DC10-K15 type, Thermoscientific, Selangor Darul Ehnsan, Malaysia). The gas transmission rate (GTR) value was determined considering the pressure increase in relation to the duration of the test and the volume of the gas. Method B with evacuation of bottom chamber was employed in the analysis, as reported in the Gas Permeability Testing Manual (2008). 100% dry pure food grade gases of O_2_ and CO_2_ were used. A mean value coming from triplicate experiments was reported.

#### 2.5.8. Thermal Analysis (Differential Scanning Calorimetry (DSC) and Thermo-Gravimetric Analysis TGA)

Differential scanning calorimetry (DSC) tests, to evaluate the characteristic temperatures and the corresponding phase change enthalpies, were carried out using a Perkin-Elmer type Pyris DSC-6 differential scanning calorimeter (Perkin-Elmer, Waltham, MA, USA), equipped with a liquid sub-ambient accessory and calibrated with high purity standards. Polymer films were cut into small pieces of about 2 mm^2^ and 10–12 mg in weight and placed in a 50 μL sealed aluminum pan. After an isotherm of 5 min at −20°C, samples were heated with a scanning rate of 10 °C/min, from −20 to 280 °C (first scan) and then, after a further isotherm of 2 min at 280 °C, were cooled to −20 °C at a rate of 80 °C/min. Finally, after an isotherm of 3 min, samples were reheated from −20 °C to 280 °C at 10 °C/min (second scan). All the experiments were performed under nitrogen flow (20 cm^3^/ min). The melting temperature (T_m_) was determined as the peak value of the endothermic phenomena in the DSC curve. The melting enthalpy (ΔH_m_) of the crystal phase was calculated from the area of the DSC endothermic peak. T_m_ and ΔH_m_ values were collected from the first scan.

Thermo-gravimetric analysis (TGA) was carried out under nitrogen atmosphere by means of Perkin Elmer TGA7 apparatus (Perkin-Elmer, Waltham, MA, USA). Gas flow of 40 mL/min and a heating scan of 10 °C/min, over a temperature range 40–800 °C, were used for the analyses. Initially, the samples were held at 40 °C for 1 min. Sample mass of 10 mg was used for the experiments.

#### 2.5.9. Attenuated Total Reflectance Infrared Spectroscopy Analysis (ATR-IR)

ATR spectra were collected on a Perkin Elmer FTIR (Perkin Elmer 1725 × Spectrophotometer, Waltham, MA, USA) over the range 650–4000 cm^−1^, with a resolution of 4.0 cm^−1^. Ten experimental tests were run on 10 different sample points and 64 scans were recorded on each sample. The experiments were performed at room temperature, directly on the samples, without any preliminary sample treatment.

## 3. Results and Discussion

### 3.1. Model Fitting

Table 1 presents the mixture design for film-forming solutions and film made from pectin, alginate and whey protein concentrate. The dependent and independent variables were fitted to linear, quadratic and cubic (special or full) models. The residual plots and normality plots were generated to verify the goodness of model. The analysis of variance was evaluated for the coefficients of the models that were significant (*p* ≤ 0.05) and with R^2^ values ≥ 0.75. Samples with a high r-squared, significant p-value and insignificant lack of fit were chosen. Following the stated parameters, the selected experimental responses (Y) and their regression coefficients (β) are presented in Table 2. In general, the positive coefficients represent synergistic effects between the components and the negative coefficients represent antagonistic effects and can be presented as an equation. The following represents special cubic model regression equation for the opacity of the films:

Y_3_ (Opacity) =3.57*P+3.35*A+0.27*WP-3.07*P*A+6.25*P*WP+12.22*A*WP+54.96*A*P*WP (6)

As in Table 2, the quadratic coefficients for responses ΔE, σ^B^, MC; special cubic coefficients for responses δ, O, WVP and full cubic model with elimination of redundant term β_23(2-3)_ for responses pH, YI, θ and E are presented, respectively. The coefficients were used to build surface plots and trace plots with sample S_1_ as a reference mixture to understand the effects of individual components on the trends of each response. The inferences drawn from response analysis as well the general effects are discussed in the subsequent sections.

### 3.2. Properties of Film forming Solutions

The obtained film forming solutions were observed to be transparent to translucent visibly, depending on their composition, with WP contributing to comparatively opaque solutions. The pH of the film-forming solutions (FFS) was measured to understand the ionic nature, as it affects the stability, functionality and characteristics of the solutions as well as of the obtained film [28]. It varied significantly ranging from 3.43 to 6.29, as reported in Table 3 (and Figure S1).

pH was observed to be influenced by the components, with alginate and whey protein increasing the value to the basic end of the scale. Pectin decreased the pH to acidic values, as expected, taking into account that commercial pectin forms solutions of pH 3.0–4.0 [28].

Total soluble solids and density were observed to be around 3.5 ( ± 0.2) Brix and 0.94 g/L ( ± 0.03) respectively, with no significant differences between tested compositions. The slight variation between values might be a function of components and their interactions. This might also be due to bonds between the hydrophilic groups of the polysaccharides with the solvent, such as water, as also observed by Zhong, Cavender and Zhao in sodium Alginate/chitosan solutions [28].

Viscosity of the film forming solution is a detrimental parameter for the leveling of coating, spread-ability, absorption by substrate, adhesion to substrate and, also, for the final film thickness, uniformity and microstructure [28,34,35]. All solutions exhibited characteristic shear thinning behavior, with decreasing viscosity as shear rates increased. For edible coatings, some authors suggested a viscosity less than 700 mPa s for casting and film forming [35]. All the film forming solutions tested were found to have viscosity in the range of 120 to 250 mPa s at a shear rate of 100s^−1^ except samples S_4_, S_6_, and S_9;_ with WP content higher than 1% w/w showing values lower than 50 mPa s. Furthermore, the rheological data were fitted to Carreau model and the parameters (a, p and η_0_) with R^2^ > 0.75 are presented in Table 3, except for S_5_ and S_6_, which fitted the power law model. The decrease in values of flow index indicated an increase in pseudo-plasticity of the fluids, and consequently an increase in consistency coefficients indicated higher apparent viscosity at a given shear rate. All the solutions exhibited higher η_0_ values and lower ‘a’ (relaxation time) values with exception of S_4_ and S_1._ These higher η_0_ values indicate strong interactions between the bio-polymers, and subsequently the lower ‘a’ (relaxation time) values, indicating the requirement of higher shear stress for structural breakdown and lower time for structural recovery, respectively. Moreover, the presence of WP tended to decrease the viscous nature of pectin and alginate, which might be attributed to the associative interactions between the polysaccharide and protein components. Similar inferences were found by Zhu, Bhandari and Prakash [29] in case of casein and whey protein isolates in combination with gelatin, pectin, and carrageen.

### 3.3. Edible Film Characterization

All obtained films were visibly transparent to translucent, flexible, and easily detachable from the petri plates, without evident defects in the form of cracks or pores. Except when only WP was used at a concentration of 3% (sample S_6_), no films were obtained as expected. As reported in literature, the formation of whey protein films usually requires an 8–12% (w/w) protein solution concentration [19,36].

#### 3.3.1. Color and Opacity

The optical properties of edible films are an essential sensory aspect for edible films and coatings to be accepted by consumers. They are generally expected to be colorless, with a transparency similar to polymeric packaging materials, or close to the food color onto which the coating will be applied [12,17]. The alginate films were more visually transparent, followed by pectin and whey protein film. Further, the films were glossier on the support side compared to the air-side, which were more dull and rough. Similar observations have been reported by Kokoszka, Debeaufort, Lenart and Voilley in their study on whey protein isolate films [37]. Regarding the color aspect, the total color difference (ΔE) and yellowness index (YI) were evaluated. Data are reported in Table 4 (and Figure S2). ΔE values ranged from 8.0 to 14.50, with P-based samples showing higher values. WP and A film showed a decreasing of values. Similar behavior was observed by Galus and Lenart [12] and Siracusa et al. [32], in edible P/A based films. Furthermore, the yellowness Index (YI) was calculated to estimate the perceived yellowness, as a low yellow value is generally preferred [31]. It was observed that the higher concentration of P and WP increased the yellowness index, probably due to the Pectin that usually favors a yellow coloration. A similar trend was found by Yasmin, Saeed, Pasha and Zia [38] in their study of pectin-whey protein beads with b* value increasing with higher pectin content.

Furthermore, the opacity (O) was observed to be ranging from 2.9 to 5.9 mm^−1^ with significant differences among samples (*p* > 0.05). As observed in literature, the presence of WP was observed to increase the opaqueness, which might be due to the formation of insoluble protein-polysaccharide aggregates during drying and due to the presence of immiscible dispersed phases in the final composite film [39,40]. Also, only a moderate correlation (r^2^ < 0.75) was observed between ΔE and opacity that might be ascribed to the interactions of components, to the network rearrangement and to the formation of aggregates during drying [41].

#### 3.3.2. Microstructure

**Figure 1:**
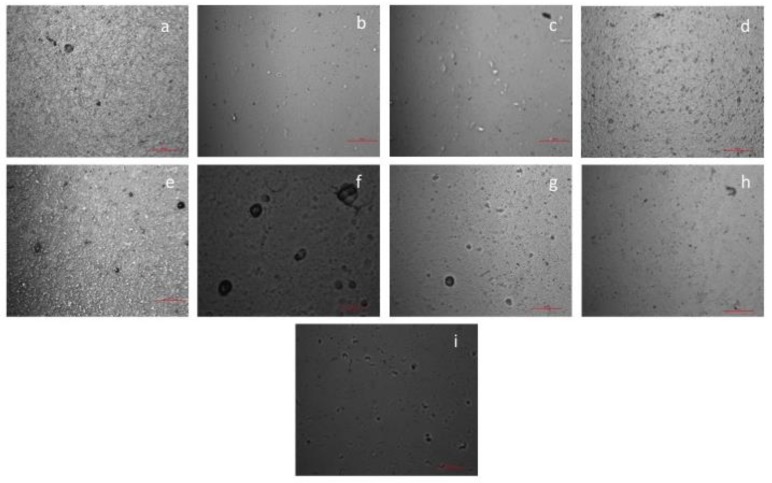
Surface micrographs of the edible films (red line 500μm): (**a**) S_1_; (**b**) S_2_; (**c**) S_3_; (**d**) S_4_; (**e**) S_5_; (**f**) S_7_; (**g**) S_8_; (**h**) S_9_; (**i**) S_10._

The micrographs of the film surfaces were acquired by optical microscopy (20× magnification) to evaluate the surface homogeneity and structure. Figure 1 shows the surface micrographs recorded. It can be seen that A-based film (S_10_) presented a more homogenous and uniform structure than P-based film (S_2_), as also observed by Siracusa et al. [32] for P/A films. However, blend films were observed to have non-uniform surfaces with formations of aggregates that can be attributed to the composition, miscibility and arrangement of the polysaccharide-protein matrices [12]. The films were noticed to be grainier and dense for secondary interactions (polysaccharide–polysaccharide, polysaccharide–protein) and with agglomerates for tertiary interactions (polysaccharide–polysaccharide–protein) which were probably formed during drying of films. These observations are in accordance with the increased opacity in the films due to protein–polysaccharide interactions as also observed by Al-Hassan and Norziah [42] and Liu, Liu, Fishman and Hicks. [43] for starch-gelatin films and pectin-gelatin films, respectively. The morphology of the films was supported by the tensile results and by gas barrier properties, evidencing how a different structural arrangement of the components in the film forming solution could affect the final behavior.

#### 3.3.3. Mechanical Properties

The thickness of composite films varied from 41 µm to 50 µm, with the exception of P (S_2_) and P: WP (S_5_) films had extreme values of 36 µm and 64 µm, respectively, as reported in Table 4. The interactions of components, as also reported by Galus and Lenart [12] were observed to affect the thickness values (*p* < 0.05) in the following order of interactions, A>P/WP>P>A/WP> P/A/WP. This variance in thickness, despite constant weight of the casting solutions, can also be attributed to the differential film drying kinetics which affect the final thickness and structure, as also observed in literature [37,44].

The mechanical properties of the edible films were measured in terms of tensile strength at break (σ^B^), elongation at break (ε^B^) and elastic modulus (E), representing the film’s mechanical resistance, plasticity and stiffness or elasticity, respectively. Generally, the tensile properties are dependent on film constituents, relative concentration and preparation conditions [32]. Films with high tensile strength in general have lower values of elongation at break, necessitating their estimation simultaneously [12,14]. This behavior was found for P (S_2_), A (S_10_) and P/A (S_3_ and S_8_) films, without or with a low concentration of the WP component, as it can be observed from the data recorded (Table 4), but not for the other films. The tensile strength values ranged from 11 MPa to 62 MPa with the highest values recorded for samples S_2_ (3:0:0) and S_3_ (1.5:1.5:0) and lowest values for S_5_ (1.5:0:1.5) and S_9_ (0.5:0.5:2) samples. P, A and the interaction of P/WP were observed to be primary factors affecting the tensile strength of the films, with P and A increasing, and P/WP interaction decreasing the tensile strength value. The interactions between A/WP, though non-significant, generally decreased the tensile strength. Similar behavior was observed in the case of elastic modulus (E), with P and A increasing the value, whereas, the linear interaction between A/WP and P/WP decreased the value. Also, these results were positively correlated (r^2^ > 0.75) to the film forming solution viscosity. This result might be due to higher available water content that acts as plasticizer, rendering the films more flexible. Indeed, lower values of elastic modulus can be considered desirable, as higher values indicate stiffer and brittle films. Subsequently, the elongation at break was affected positively by the interaction between A/WP, causing an increase in ε^B^, indicative of higher elasticity. The decrease in tensile strength and elastic modulus upon addition of whey protein, was similar to that of whey protein isolate and whey protein concentrate films observed by Ramos et al. [45].

#### 3.3.4. Moisture Content (MC), Water Vapor Permeability (WVP) and Water Contact Angle (WCA)

The moisture content of the films was measured to estimate the water bonding capacity of the films. The moisture content of the tested films ranged from 6.6 to 13.7% (Table 5 and Figures S3–S5), with significant influence of WP concentration and linear interactions (like between P/A, P/WP or A/WP). The hygroscopic nature of glycerol favors the absorption of water molecules, and the formation of hydrogen bonds in a film matrix, which lead to the higher moisture content of the films as in the S_4_ sample [13]. However, the moisture content values for all the films were observed to be lower than those presented for pectin, alginate or whey protein films reported in literature [45].

Water vapor permeability for the tested films ranged from 7.2 to 9.6 (10^10^g mm h−^1^ cm^−1^ Pa^−1^), for different formulations (Table 5). P and A-based films recorded lower values, whereas WP presence slightly increased the permeability values, which could be attributed to the higher number of free hydroxyl groups, thereby enhancing interaction with water and favoring the water vapor transmission. Also, this is dependent on the number of polar groups the polymer contains, and on the diffusivity and solubility of water molecules in the film matrix [37,46]. However, it was interesting to note that the water barrier property of high WP containing films might be attributed to the presence of lactose that exerts plasticizing effect on the polymer, facilitating the water transfer. In accordance to the moisture results, the WP was found to have significant effect on the barrier property.

An opposite effect was observed concerning the surface hydrophobicity of the films, with P and A decreasing and WP increasing the WCA values’ rise, as reported in Table 5 (and Figure S4). As is well known, a water contact angle (θ) θ < 65 indicates hydrophilic character, while θ > 65 represents hydrophobic character [46,47]**.** WCA value was observed to be slightly higher on the air-side than on the support side of the films (data not shown) as also observed in a study on Chitosan and whey protein films [22]. The lowest value was observed in P-based films, whereas, the addition of A and WP significantly increased the WCA from 40° to around 50° and >60°, respectively (Table 5). The significant lack of fit with full cubic model was due to low pure error; however, it did not affect the predicting ability of the model. A, A/WP, P, P/WP, P/A/WP had significant positive influences whereas, the interaction terms were found to negatively affect this response. Furthermore, a significant positive correlation (r^2^ > 0.75) was observed between those three parameters confirming the increase in moisture content leads to increased water vapor permeability and hydrophilicity of the films.

#### 3.3.5. Gas Barrier Properties

**Figure 2:**
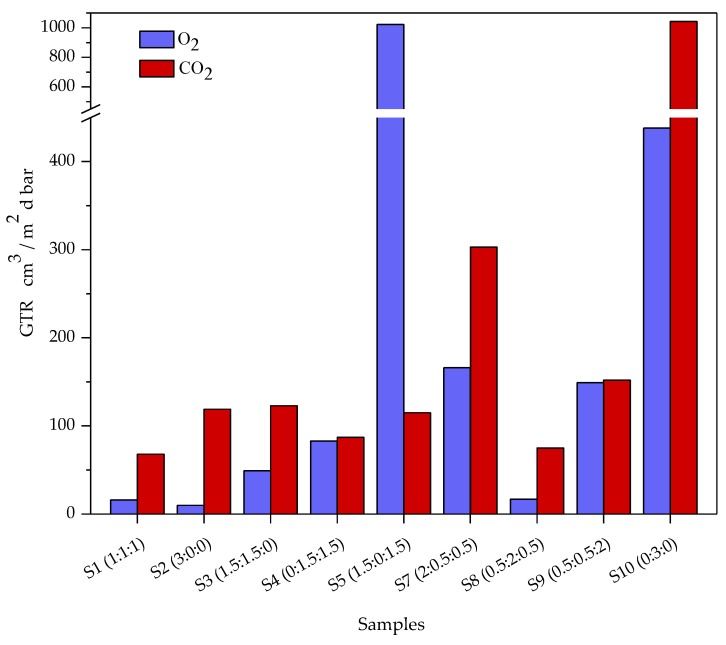
Gas transmission rate (GTR) recorded at 23°C, with an O_2_ and CO_2_ gas test, for edible films samples.

As is well known, odor, color, flavor and nutrient content of food are strongly influenced by oxidation and environmental contact. Further, food quality and shelf life depend on the film ability retarding oxidation and degradation processes of the products. Therefore, gas barrier properties are fundamental in order to preserve food from any modification. Gas barrier properties of films for gases, particularly, O_2_ and CO_2_ are important as they affect the respiration or oxidation reactions in foods and are useful to identify niche applications. As is well known [32], those properties are strongly dependent on the polymer structure, gas type and temperature. In this contest, edible films barrier were examined at 23 °C (room temperature), with 100% pure food grade O_2_ and CO_2_ and data recorded are reported in Table 6 and in Figure 2. The gas transmission rate (GTR) was found to be significantly affected by the composition of the films. The highest gas permeation was recorded for pure alginate film, despite its higher enthalpy value and more homogeneous and uniform structure, with both gases. The pure pectin film showed a lower permeability behavior, despite its lower enthalpy value, due to its homogeneous and uniform structure and its higher rigidity, as described before. The addition of WP in general decreased the transmission rates [48] explained by a good correlation with thermal and mechanical data, although the film exhibited heterogeneous behavior when observed under optical microscope. Gas permeability through matrices is complex, because it depends on different parameters. Some are related to the gas characteristics such as size, polarity, inertness toward materials and so on; others are linked to the material properties, such as degree of crystallinity, presence of cross-linking, chain stiffness, and so on. In general, the more tortuous is the gas path, the lower is the gas transmission rate [49]. Also, as often observed in literature and as observed for other similar polymers previously investigated [32,50,51], CO_2_ permeation is in general higher compared to that of O_2_, due to diffusivity drop and solubility incrementation with decreasing permeant size (molecular diameter of CO_2_ 3.4 Å and O_2_ molecular diameter 3.1 Å, respectively) [52]. The presence of a hydrophopic matrix contributes to reduce the solubility of carbon dioxide, increasing the GTR value [49]. However, it is worth noting that the gas transmission rates to oxygen and carbon dioxide were not so different in same samples, due to the higher affinity (high solubility) of CO_2_ gas molecules within the matrix.

The gas perm-selectivity ratio (CO_2_/O_2_), which represents the permeability ratio of different permeants, is also reported in Table 6. Those values are useful because they allow the calculation of the unknown GTR, knowing the GTR value of another gas. As reported in the literature [53], for many polymers the N_2_:O_2_:CO_2_ is in the range of 1:4:16. Our results were very similar to those tabulated in the literature, demonstrating that using these values for calculations would be appropriate. But, as previously demonstrated [54,55], those ratios are not always constant, but depend on the chemical structure, temperature, moisture and so on, with behavioral deviation. Our ratio was relatively low for all films, with a value below 5.0, equivalent to synthetic polymers, with the exception for sample S_1_ with a value of 11.5 and for sample S_2_ (pure Pectin) with a value of 12.4, higher than those observed by Siracusa et al. [32], for the reason explained above.

#### 3.3.6. Thermal Properties

The effect of protein interaction with polysaccharides on the thermal properties, such as melting temperature (T_m_, °C) and enthalpy of fusion (ΔH_m_, J/g), were measured by DSC. The data of the first scan are reported in Table 7. The heat capacity associated to the glass-to-rubber transition was too low to determine the T_g_ values. The T_m_ of P-based film (98 °C) was observed to be significantly lower than the A-based film (124 °C) and composite films, as also observed from Ramos et al. on whey protein based edible films [45]. Further, pure alginate films (S_2_) showed melting enthalpy higher than the pure pectin film (S_10_), as evicted also from the results on microstructure. The composite films showed T_m_ values in the range of 116 °C to 133 °C with WP presence increasing the T_m_ position upon interaction with P. The presence of A enhances the enthalpy values, indicative of increased crystallinity as a consequence of inter chain interactions. The S_1_ film, containing the same amount of all three components P/A/WP presents the highest values both of T_m_ and ΔH_m_, indicative of great miscibility between components.

**Figure 3:**
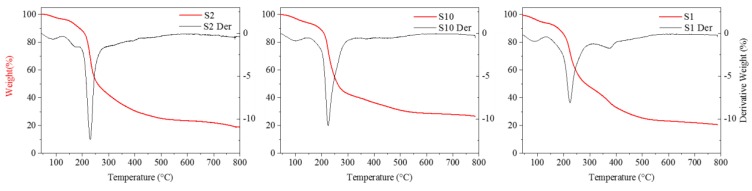
Representative thermo gravimetic analysis (TGA) spectra for pectin (S_2_), alginate (S_10_) and P/A/ WP (S_1_) films.

The thermal stability of the films was analyzed by TGA [56]. Figure 3 depicts representative curves of residual mass and the corresponding first derivatives. All TGA traces show an initial weight loss below 130 °C due to the adsorbed and bound water [57,58]. Nevertheless, all the films studied exhibited good thermal stability up to 200 °C. The degradation onset was between 175 °C to 221 °C and the maximum degradation rate was between 217 °C to 235 °C. The weight loss was observed to be sharper in pure pectin and alginate films. In the tertiary blend films, the thermal degradation was more gradual, starting at slightly lower temperatures than pure films, as reported in Table 7. The final residue at 600 °C was observed to be between 22–25% except for pure alginate film which was of 29%. The observed results are in accordance to those of Ramos et al. [45] on WPC films and Siracusa et al. [32] on alginate-pectin films.

#### 3.3.7. Infrared Spectroscopy

Near infrared spectroscopy (NIR) was previously used in order to study the drying process of edible coating on bread surface [23].

**Figure 4:**
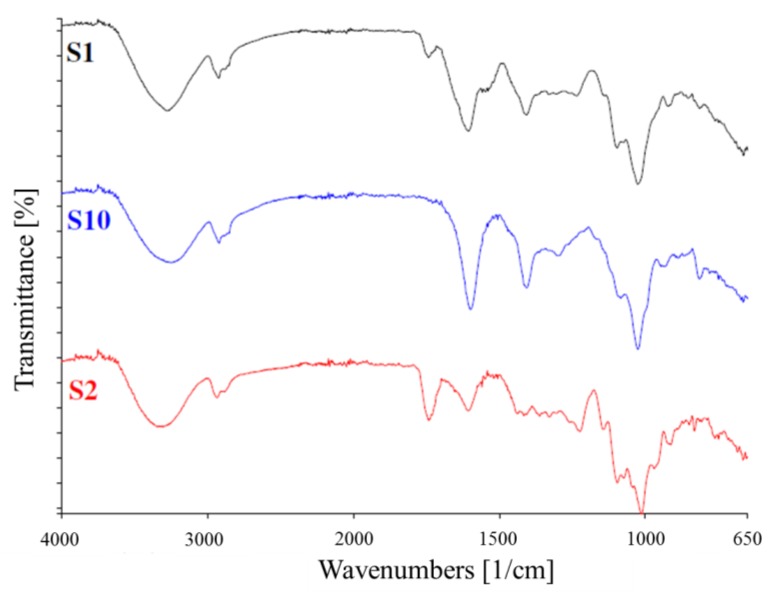
Representative FTIR Spectral patterns of pectin (S_2_), alginate (S_10_) and P/A/WP (S_1_) films.

The FTIR spectra of the films with various mixtures of P/A/WP are displayed in Figure 4.

The pure pectin film was characterized by a broad band at 3343cm^−1^ (O-H stretch); a small peak and a shoulder at 2934 cm^−1^ and 2884 cm^−1^ (C–H vibrations of methyl esters); 1744 cm^−1^ (C=O bonds of esters); 1607 cm^−1^ and 1416 cm^−1^ (the carboxyl symmetric and asymmetric vibrations, respectively); and the typical saccharide finger print region from 800 cm^−1^ to 1300 cm^−1^. The pure alginate film was characterized by a broad band at 3252 cm^−1^ (O–H stretch); a small peak and a shoulder at 2920 cm^−1^ and 2853 cm^−1^ (asymmetric C–H vibrations); 1599 cm^−1^ and 1407 cm^−1^ the carboxyl asymmetric vibrations and C–OH deformation vibrations, respectively; and the typical saccharide from 800 cm^−1^ to 1300 cm^−1^. However, given the case of pure WP, films were not characterized and literature was used to identify the probable interactions. For the blend films, the total number and the intensities of the peaks varied based on their compositions. All the blend films exhibited the typical peaks with respect to the pectin and alginate. A marked difference is the presence of an additional peak at 1530-1550 cm^−1^ in films with higher whey protein, indicative of the N–H bend of amide II. Also, in these films’ small peaks at 1200–1230 cm^−1^ were observed indicative of the C–N stretch vibrations of amide III. However, the C–N stretch typical at 1600–1700 cm^−1^ was camouflaged by the -COO symmetric vibrations and only a shift in wave numbers can be observed.

The identification of the characteristic peaks allowed confirmation for all films of the expected structure, as reported by Siracusa et al. [32] on citral essential oil added as an antioxidant and antimicrobial agent for pectin-alginate edible film.

## 4. Conclusions

The screening of an edible film composition is important because it must be formulated to maintain or improve the food quality. The design of mixtures was found to be a useful tool to study the effect of component concentrations on the properties of the film forming solutions and edible films. Physico-chemical and structural analyses have been performed on the prepared formulations to evaluate the properties of interest for the potential application on various products. In general, films appeared homogeneous and transparent, but the effect of the components on the mechanical properties of edible films is quite complex, as well as their gas barrier behavior, since both enhancing and weakening of the characteristics have been observed. Specific interactions between the film components, different structural arrangements, or formation of heterogeneous biphasic structures must be considered. Whey protein was observed to have significant effects on the physico-chemical, rheological properties of the solutions, as well as the optical and mechanical properties of the films formed. The increase in whey protein contributed to lowering the viscosity but significantly favored hydrophobicity of the films, causing a reduction of the water absorption that can act as plasticizer. The best performances in term of thermal, mechanical and gas barrier behavior were observed for lower P and WP concentration and higher A amount, or when the same P/A/WP content was used even though with similar thermal degradation patterns. The presence of WP was observed to play a beneficial role in decreasing the wettability of the edible film, making them suitable for food wrapping applications. Considering the synergistic effects the tertiary blends of P/A/WP have scope to be further optimized to provide blends with improved barrier and mechanical properties.

The investigated films could be taken into consideration as potential natural packaging materials for improve the shelf life of bakery food.

## Figures and Tables

**Table 1:** Composition of the film forming solutions formulated with pectin, alginate and whey protein concentrate by simplex centroid mixture design.

Sample	% W/W		
	P	A	WP
S_1_	1.00	1.00	1.00
S_2_	3.00	0.00	0.00
S_3_	1.50	1.50	0.00
S_4_	0.00	1.50	1.50
S_5_	1.50	0.00	1.50
S_6_	0.00	0.00	3.00
S_7_	2.00	0.50	0.50
S_8_	0.50	2.00	0.50
S_9_	0.50	0.50	2.00
S_10_	0.00	3.00	0.00

P- Pectin, A- Alginate, WP- Whey protein concentrate.

**Table 2:** Regression coefficients of the response variables for pseudo-components and analysis of variance for fitted models.

Term	Response variables									
	Y_1_ (pH)	Y2 (δ)	Y3 (O)	Y_4_ (ΔE)	Y_5_ (YI)	Y_6_ (θ)	Y_7_ (σ^B^)	Y_8_ (E)	Y_9_ (WVP)	Y_10_ (MC)
P (β_1_)	3.43 **	34.32 **	3.57 **	11.26 **	9.63 **	40.21 **	58.25 **	1473.6 **	1.59 **	8.97 **
A(β_2_)	5.83 **	40.70 **	3.35 **	8.75 **	4.31 **	55.40 **	39.14 **	606.9 **	1.92 **	6.33 **
WP (β_3_)	6.21 **	2.81 ^n.s^	0.27 ^n.s^	0.73 ^n.s^	0.05 ^n.s^	0.38 ^n.s^	2.60 ^n.s^	0.3 ^n.s^	0.12 ^n.s^	0.44 ^n.s^
P X A (β_12_)	−2.07 **	11.99 ^n.s^	−3.07 ^n.s^	12.94 *	2.40 ^n.s^	15.76 ^n.s^	20.54 ^n.s^	−327.9 ^n.s^	−0.88 ^n.s^	-
P X WP (β_13_)	−1.40 **	185.94 **	6.25 ^n.s^	7.49 ^n.s^	30.87 **	163.75 **	−87.07 *	−1504.5 **	7.09 **	20.23 **
A X WP (β_23_)	1.03 **	107.07 **	12.22 **	15.08 *	38.10 **	206.07 **	−5.23 ^n.s^	413.4 ^n.s^	4.02 **	36.04 **
P X A X WP (β_123_)	−3.31 ^n.s^	−398.45 *	54.96 *	-	−76.88 **	291.07 **	-	−2410.9 ^n.s^	−14.69 *	-
β_12(1-2)_	5.08 *	-	-	-	4.01 ^n.s^	−8.90 ^n.s^	-	−3062.9 **	-	-
ß_23(2-3)_	-	-	-	-	-	-	-	-	-	-
β_13(1-3)_	0.06 ^n.s^	-	-	-	−31.54 **	−316.54 **	-	−2043.4 *	-	-
Model-p	0.00	0.00	0.00	0.00	0.00	0.00	0.00	0.00	0.00	0.00
R^2^	0.99	0.88	0.79	0.84	0.97	0.99	0.86	0.98	0.87	0.81
Lack of fit	0.41	0.12	0.09	0.09	0.29	0.00	0.12	0.92	0.14	0.01
										

δ = thickness; O=opacity; ΔE=total colour difference; YI = Yellowness index; θ = Water contact angle; σ^B^ = Tensile strength at break; E= Elastic modulus; WVP = Water vapour permeability; MC = Moisture content. * terms sigificant at 0.05 level. ** terms significant at 0.001 level. ^n.s^ non-signifiant terms, included for trend analysis.

**Table 3:** Physico-chemical and rheological properties of the edible coating solution.

Sample (P/A/WP)	Carreau Parameters			
	pH	P	A	η (mPa s)
S_1_ (1:1:1)	4.80 ± 0.01 ^d^	0.10 ± 0.00 ^de^	0.24 ± 0.00 ^cd^	247.0 ± 1.27 ^cd^
S_2_ (3:0:0)	3.43 ± 0.01 ^a^	0.08 ± 0.01 ^cb^	0.02 ± 0.00 ^ab^	272.0 ± 7.00 ^d^
S_3_(1.5:1.5:0)	4.12 ± 0.01 ^b^	0.07 ± 0.00 ^abc^	0.05 ± 0.008 ^c^	249.0 ± 1.53 ^cd^
S_4_(0:1.5:1.5)	6.29 ± 0.03 ^h^	0.07 ± 0.01 ^ab^	0.01 ± 0.00 ^a^	46.2 ± 2.26 ^a^
S_5_(1.5:0:1.5)	4.48 ± 0.04 ^c^	*	*	*
S_6_(0:0:3)	6.22 ± 0.06 ^h^	*	*	*
S_7_(2:0.5:0.5)	4.14 ± 0.03 ^b^	0.05 ± 0.00 ^a^	0.05 ± 0.01 ^c^	154.0 ± 7.83 ^b^
S_8_(0.5:2:0.5)	4.98 ± 0.1 ^e^	0.08 ± 0.00 ^bd^	0.02 ± 0.00 ^a^	143.0 ± 2.47 ^b^
S_9_(0.5:0.5:2)	5.50 ± 0.01 ^f^	0.05 ± 0.00 ^a^	0.20 ± 0.06 ^cd^	45.1 ± 0.88 ^a^
S_10_(0:3:0)	5.84 ± 0.03 ^g^	0.09 ± 0.01 ^de^	0.02 ± 0.00 ^ab^	224.0 ± 6.95 ^c^

Means ± SD in a column without a common superscript letter differ significantly (*p* < 0.05). η_0_ = viscosity at low shear rate, a = characteristic time constant, p = dimensionless Carreau exponent. * Sample 5 (P:A:WP = 1.5:0:1.5) and Sample 6 (P:A:WP = 0:0:3) were modeled using a power-law equation, as they did not fit Carreau model. The theoretical values of ‘k’ (consistency index, Pa.s^−n^) and ‘n’ (flow behavior index, dimensionless) were k = 0.912 and n = −0.352 for S_5_ and k = 0.002 and n = 1.00 for S_6_, respectively.

**Table 4:** Physical and mechanical properties of the edible films.

Sample	δ (µm)	ΔE	YI	O	E (MPa)	σ^B^ (MPa)	ε^B^ (%)
S_1_ (1:1:1)	45 ± 4 ^b^	8.6 ± 0.4 ^a^	9.3 ± 0.9 ^cd^	6.0 ± 0.2 ^f^	444 ± 107 ^abc^	23 ± 6 ^abc^	22 ± 9
S_2_ (3:0:0)	36 ± 1 ^a^	11.3 ± 1.1 ^b^	9.6 ± 1.1 ^cd^	3.4 ± 0.1 ^bc^	1467 ± 303 ^d^	62 ± 20 ^d^	20 ± 8
S_3_ (1.5:1.5:0)	43 ± 2 ^b^	14.1 ± 1.0 ^c^	7.5 ± 0.4 ^b^	2.9 ± 0.1 ^a^	958 ± 240 ^d^	61 ± 11 ^d^	23 ± 10
S_4_ (0:1.5:1.5)	47 ± 1 ^bc^	8.5 ± 0.5 ^a^	11.6 ± 0.5 ^ef^	5.0 ± 0.2 ^e^	406 ± 145 ^ab^	22 ± 9 ^abc^	40 ± 23
S_5_ (1.5:0:1.5)	64 ± 2 ^d^	8.1 ± 0.6 ^a^	12.5 ± 0.9 ^f^	3.0 ± 0.1 ^ab^	352 ± 140 ^ab^	13 ± 4 ^ab^	16 ± 7
S_7_ (2:0.5:0.5)	43 ± 2 ^b^	11.5 ± 0.9 ^b^	9.2 ± 1.3 ^cd^	5.3 ± 0.2 ^e^	555 ± 129 ^abc^	27 ± 5 ^abc^	26 ± 4
S_8_ (0.5:2:0.5)	41 ± 1 ^b^	11.42 ± 1.0 ^b^	8.5 ± 0.5 ^bc^	4.1 ± 0.3 ^d^	746 ± 127 ^cd^	31 ± 10 ^bc^	24 ± 12
S_9_ (0.5:0.5:2)	49 ± 1 ^c^	8.8 ± 1.0 ^a^	10.7 ± 0.4 ^de^	5.2 ± 0.2 ^e^	288 ± 59 ^a^	11 ± 3 ^a^	19 ± 2
S_10_ (0:3:0)	42 ± 1 ^b^	8.6 ± 0.8 ^a^	4.3 ± 0.1 ^a^	3.7 ± 0.4 ^cd^	607 ± 243 ^bc^	39 ± 16 ^c^	27 ± 14

Means ± SD in a column without a common superscript letter differ significantly (*p* < 0.05). δ = thickness; ΔE = total colour difference; YI = Yellowness index; O = opacity; E = Elastic modulus; σ^B^ = Tensile strength at break; ε^B^ = elongation at break.

**Table 5:** Moisture content (MC), water vapor permeability (WVP) and Water contact angle (WCA) of composite films.

Sample (P/A/WP)	MC (%)	WVP (10^10^ g mm h^−1^ cm^−1^ Pa^−1^)	WCA (θ)
S1 (1:1:1)	12.2 ± 1.7 ^cd^	7.9 ± 0.4 ^ab^	83 ± 4.1 ^de^
S_2_ (3:0:0)	8.6 ± 1.1 ^ab^	7.3 ± 0.4 ^a^	40 ± 2.1 ^a^
S_3_ (1.5:1.5:0)	6.6 ± 1.3 ^a^	7.4 ± 0.3 ^a^	51 ± 1.4 ^b^
S_4_ (0:1.5:1.5)	13.7 ± 1.4 ^d^	8.7 ± 1.1 ^ab^	79 ± 0.9 ^d^
S_5_ (1.5:0:1.5)	11.9 ± 0.6 ^cd^	9.6 ± 0.5 ^b^	60 ± 2.5 ^c^
S_7_ (2:0.5:0.5)	6.8 ± 1.1 ^a^	7.9 ± 0.2 ^ab^	51 ± 1.5 ^b^
S_8_ (0.5:2:0.5)	7.4 ± 0.5 ^a^	7.5 ± 0.4 ^a^	81 ± 1.2 ^de^
S_9_ (0.5:0.5:2)	10.8 ± 1.3 ^bc^	9.0 ± 0.1 ^b^	83 ± 2.1 ^e^
S_10_ (0:3:0)	8.5 ± 1.6 ^a^	7.2 ± 0.1 ^a^	55 ± 1.1 ^b^

Means ± SD in a column without a common superscript letter differ significantly (*p* < 0.05).

**Table 6:** Oxygen and carbon dioxide permeability data for edible films.

Sample (P/A/WP)	O_2_ (cm3 m-2d^−1^ bar^−1^)	CO_2_ (cm3 m-2d^−1^ bar^−1^)	CO_2_/O_2_
S_1_ (1:1:1)	16 ± 21 ^f^	68 ± 2 ^ab^	4.3
S_2_ (3:0:0)	10 ± 2 ^a^	119 ± 2 ^ab^	12.4
S_3_ (1.5:1.5:0)	49 ± 3 ^a^	123 ± 2 ^a^	2.5
S_4_ (0:1.5:1.5)	83 ± 1 ^c^	87 ± 2 ^a^	1.0
S_5_ (1.5:0:1.5)	1023 ± 3 ^b^	115 ± 4 ^ab^	11.5
S_7_ (2:0.5:0.5)	166 ± 1 ^d^	303 ± 6 ^c^	1.8
S_8_ (0.5:2:0.5)	17 ± 3 ^c^	75 ± 4 ^a^	4.4
S_9_ (0.5:0.5:2)	149 ± 4 ^d^	152 ± 4 ^b^	1.0
S_10_ (0:3:0)	438 ± 16 ^e^	1043 ± 59 ^d^	2.4

Means ± SD in a column without a common superscript letter differ significantly (*p* < 0.05).

**Table 7:** Differential scanning calorimetry and thermogravimetric data for edible films.

Sample (P/A/WP)	Degradation Onset (°C)	Degradation Peak (°C)	T_m_ (°C)	ΔH_m_ (J g^−1^)
S_1_ (1:1:1)	207 ± 10 ^a^	224 ± 1 ^a^	130 ± 1 ^b^	124 ± 5 ^cd^
S_2_ (3:0:0)	220 ± 4 ^a^	231 ± 2 ^a^	98 ± 7 ^a^	89 ± 8 ^ab^
S_3_ (1.5:1.5:0)	205 ± 3 ^a^	217 ± 1 ^a^	133 ± 2 ^b^	112 ± 11 ^abcd^
S_4_ (0:1.5:1.5)	222 ± 1 ^a^	235 ± 1 ^a^	121 ± 7 ^b^	122 ± 10 ^cd^
S_5_ (1.5:0:1.5)	212 ± 12 ^a^	229 ± 1 ^a^	125 ± 6 ^b^	93 ± 6 ^abc^
S_7_ (2:0.5:0.5)	202 ± 0 ^a^	222 ± 0 ^a^	127 ± 8 ^b^	81 ± 17 ^a^
S_8_ (0.5:2:0.5)	208 ± 3 ^a^	222 ± 1 ^a^	127 ± 2 ^b^	139 ± 11 ^d^
S_9_ (0.5:0.5:2)	175 ± 6 ^a^	225 ± 3 ^a^	116 ± 8 ^b^	114 ± 10 ^abcd^
S_10_ (0:3:0)	211 ± 2 ^a^	225 ± 4 ^a^	124 ± 7 ^b^	117 ± 19 ^bcd^

Means ± SD in a column without a common superscript letter differ significantly (*p* < 0.5).

## Supplementary Materials

The following are available online at https://www.mdpi.com/1996-1944/12/15/2454/s1: Figure S1: 3D surface plot of the effects of components on pH of FFS where the region of A-WP interaction increased the solution pH and Pectin decreased the pH; Figure S2: 3D surface plot of the effects of components on opacity of edible films where the interaction of WP with P/A increased the opaqueness; Figure S3: 3D surface plot of the effects of components on water contact angle (θ) of edible films where the blending of WP/P/A results in higher convexity indicating improved hydrophobcity of films; Figure S4: 3D surface plot of the effects of components on moisture content (%) of edible films; Figure S5: Representative plots of viscosity (vs.) shear rate for selected formulations S1 (1:1:1); S2 (3:0:0); S6(0:0:3) and S10(0:3:0).
