# Supplementary material for: Characterization of Composite Edible Films Based on Pectin/Alginate/Whey Protein Concentrate
Source: Materials (Basel). 2019 Aug 1;12(15):2454. doi: 10.3390/ma12152454 (PMC6696009; doi:10.3390/ma12152454)
Supplement: Supplementary file 1 [file materials-12-02454-s001.pdf]

materials

Article

Characterization of Composite Edible Films Based on Pectin/Alginate/Whey Protein Concentrate

Swathi Sirisha Nallan Chakravartula [1], Michela Soccio [2], Nadia Lotti [2], Federica Balestra [1], Marco Dalla Rosa [1] and Valentina Siracusa [3,*]

[1] Department of Agricultural and Food Sciences- DISTAL, University of Bologna, Campus of Food Science, P.zza Goidanich 60, 47521 Cesena, Italy; swathisirisha.nalla2@unibo.it (S.S.N.C.); federica.balestra@unibo.it (F.B.); marco.dallarosa@unibo.it (M.D.L.)

[2] Department of Civil, Chemical, Environmental and Materials Engineering, University of Bologna, Via Terracini 28, 40131 Bologna, Italy; nadia.lotti@unibo.it (N.L.), m.soccio@unibo.it (M.S.)

[3] Department of Chemical Science, University of Catania, Viale A. Doria 6, 95125 Catania (CT), Italy; vsiracus@dmfci.unict.it (V.S.)

* Correspondence:Correspondence: vsiracus@dmfci.unict.it; Tel.: +39-338-727-5526, (V.S.)

Received: 26 June 2019; Accepted: 30 July 2019; Published: date

The following is supplementary data providing the 3D response plots for the selected responses from table 2.

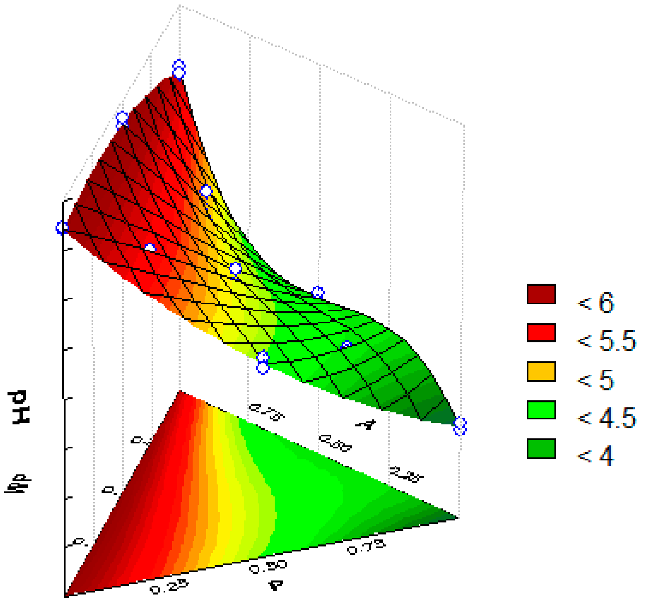

Figure S1. 3D surface plot of the effects of components on pH of FFS where the region of A-WP interaction increased the solution pH and Pectin decreased the pH.

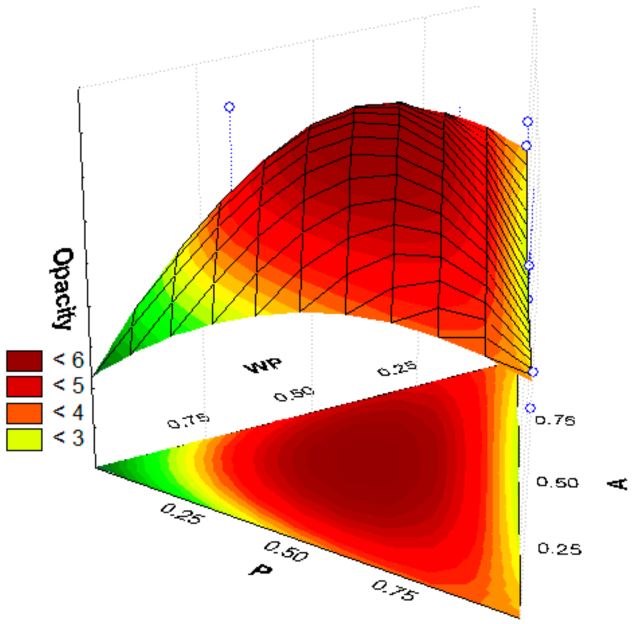

Figure S2. 3D surface plot of the effects of components on opacity of edible films where the interaction of WP with P/A increased the opaqueness.

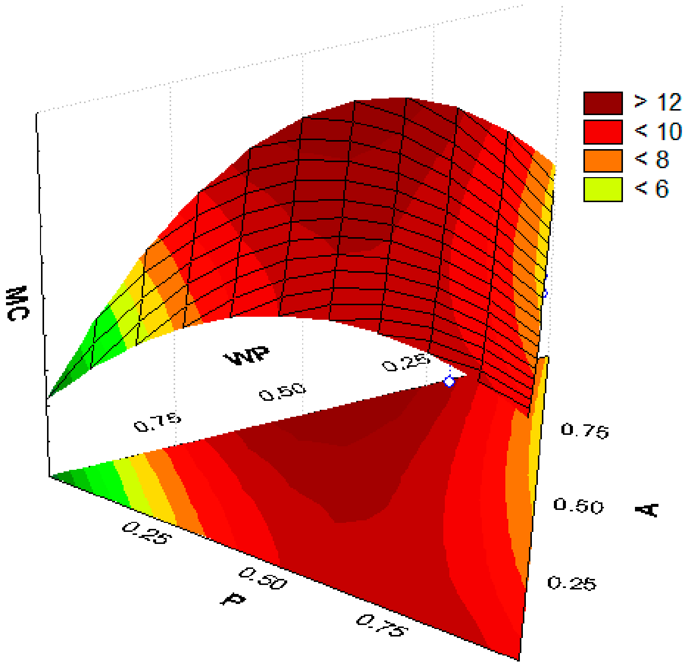

Figure S3. 3D surface plot of the effects of components on Moisture content (%) of edible films.

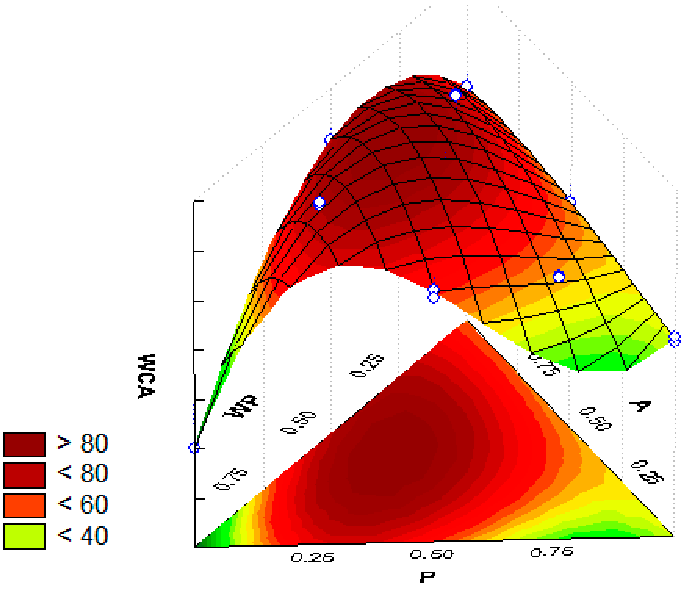

Figure S4. 3D surface plot of the effects of components on water contact angle (θ) of edible films where the blending of WP/P/A results in higher convexity indicating improved hydrophobcity of films.

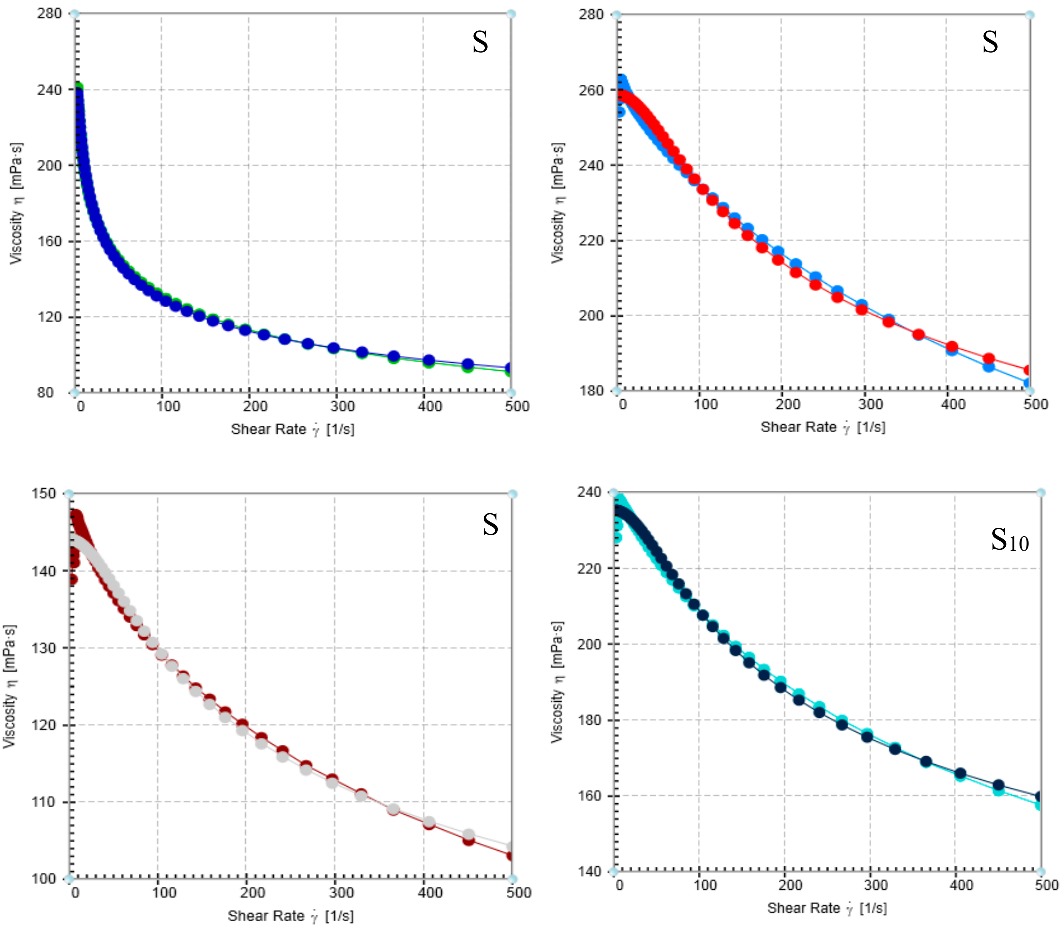

Figure S5. Representative plots of viscosity (vs.) shear rate for selected formulations S1 (1:1:1); S2 (3:0:0); S6(0:0:3) and S10(0:3:0).
